# Interaction between Geographical Areas and Family Environment of Dietary Habits, Physical Activity, Nutritional Knowledge and Obesity of Adolescents

**DOI:** 10.3390/ijerph20021157

**Published:** 2023-01-09

**Authors:** Alessio Pellegrino, Samuele Bacci, Francesco Guido, Andrea Zoppi, Loira Toncelli, Laura Stefani, Maria Boddi, Alessandra Modesti, Pietro Amedeo Modesti

**Affiliations:** 1 Sport Medicine Unit, Department of Experimental and Clinical Medicine, University of Florence, 50134 Florence, Italy; 2 Department of Biomedical, Experimental and Clinical Sciences “Mario Serio”, University of Florence, 50134 Florence, Italy

**Keywords:** family environment, obesity, physical activity, dietary habits, adolescents, nutritional knowledge, parental influence

## Abstract

There are marked differences in the regional distribution of childhood obesity in Italy. This study sought to investigate the interaction between geographical areas and family environment of dietary habits, physical activity, nutritional knowledge and obesity of adolescents. A cross-sectional study was conducted on 426 school-aged children and 298 parents residing in Central Italy (Florence, Tuscany) and Southern Italy (Corigliano, Calabria), in 2021. Survey questionnaire investigated anthropometry, eating behavior, nutritional knowledge and physical activity. BMI was determined and compared with reference percentile charts for adolescents. Multivariate regression analyses showed that: (1) an adolescent’s BMI was directly influenced by their parents’ BMI independently of parental nutritional knowledge and dietary or physical activity habits; (2) parents transmitted eating or physical activity habits to their children; (3) the geographic region of residence is not in itself an independent determinant of children’s BMI. The clear message is that prevention of childhood obesity should consider family-based approaches. Parental obesity can be the point of convergence of the complex interactions between a parent’s and child’s habits and should be one of the most important factors to look for.

## 1. Introduction

In recent decades, childhood obesity has grown globally and is now a major public health problem [1]. In 2016, over 50 million girls and 70 million boys were obese at a global level [2]. Excess body fat has been attributed to both a high intake of energy-dense, nutrient-poor foods and beverages, such as chips, salted snacks, sweets and soft drinks, and a sedentary lifestyle [3]. In Europe, a North–South obesity gradient has been observed. Italy, particularly the Southern region of the country [4], has one of the highest prevalences of childhood overweight and obesity in the European WHO region [5]. Growing evidence indicates that environmental and socio-economic factors play a relevant role among determinants of the childhood obesity epidemic. Parental habits can influence children’s habits early in life [6,7]. Growth from childhood to adolescence is strongly influenced by the interaction between genetic [8], social, nutritional and environmental factors, that increasingly come from the world outside the family, and in particular from school and the community [9]. Therefore, the study of the interaction between family or environmental factors and body weight is important to identify determinants [10] and potential intervention targets for childhood obesity [11].

The present study sought to investigate the influence of parental physical activity, dietary habits, obesity level, sociocultural factors and the interaction with the geographic area, on the weight status of children and adolescents in Italy.

## 2. Materials and Methods

### 2.1. Design and Study Population

A cross-sectional web-based observational epidemiological study was conducted in 2021 on students of primary or secondary schools and their parents in Florence (Tuscany, located in Central Italy) and in Corigliano (Calabria, Southern Italy). Eligible subjects were residents of the Florence or Corigliano area with no plans to move within the study period. Exclusion criteria included comorbidities that required immediate medical attention, use of medication affecting body weight, diagnosis of Type 1 diabetes or participation in a weight-loss program.

### 2.2. Ethics

The experimental protocols and the process for obtaining informed consent were approved by the Institutional Review Board (Prot. n. 0084484) in conformity with the principles embodied in the World Medical Association Declaration of Helsinki and with the general data protection regulations. Participation in the study was voluntary. Participants expressed their informed consent prior to filling in the questionnaire.

### 2.3. Study Procedure

Preliminary informative web meetings were held with school teachers, students and their parents. Participants were then invited to complete an anonymous but coded (to associate children with parents) web-based questionnaire. The questionnaire was composed of 4 main sections investigating (a) demography and anthropometry, (b) diet composition (Mediterranean Diet Score assessment) [12], (c) physical activity levels (International Physical Activity Questionnaire, IPAQ) [13] and nutritional knowledge (the General and Sport Nutrition Knowledge questionnaire, GeSNK) [14].

*a. Demography and anthropometry.* This section included questions regarding age (years), gender, parent education level (Secondary school, High school, University), weight (kg) and height (cm). Body mass index (BMI) was then calculated. Underweight, normal weight, overweight or obese were coded according to WHO BMI-for-age standard percentiles [15,16] for students or WHO standards [15,17] for parents.

*b. Diet composition* was investigated using the Mediterranean Diet (MD) score assessment questionnaire [12] as previously reported [18]. Briefly, the weekly consumption of 9 food groups (unrefined cereals such as wholemeal bread, wholemeal pasta, rice, etc.; potatoes; fruit; vegetables; legumes; fish; red meat; white meat; dairy products including cheese, yogurt, milk), as well as olive oil and alcohol intake were investigated. The resulting index of adherence ranged between 0 and 55 points [12].

*c. Physical Activity Levels* were investigated using the International Physical Activity Questionnaire—short form (IPAQ-SF) validated questionnaire [19,20,21]. Briefly, the total volume of leisure time physical activity accumulated over a week, expressed as metabolic equivalents (METs) h/week, was assessed and participants were categorized by compliance to WHO criteria for physical activity as previously reported [18]. Subjects were also classified as: inactive (<700 METs h/wk.); sufficiently active (700–2519 METs h/wk.); active or very active (>2519 METs h/wk.) [22].

*d. Nutrition knowledge* was investigated using the General and Sport Nutrition Knowledge (GeSNK) validated questionnaire [14]. The GeSNK questionnaire included 62 items divided in 2 different sections (see the questionnaire in the Supplementary Materials). Specifically, the first section (items 1–29) is concerned with general nutrition (GeSNK general nutrition); the second section (items 30–62) is concerned with nutrition and sport (GeSNK nutrition and sport). The majority of the questions could be answered ‘True’, ‘False’ or ‘I don’t know’. Scores were coded as +1 for a correct answer and 0 if participants selected the incorrect answer or the ‘I don’t know’ response. The maximum total score was 97 and the minimum was 0. The knowledge of participants who obtained a score lower than 46 and higher than 58 was labelled as ‘low’ or ‘high’ knowledge, respectively. An intermediate score was considered ‘medium’ [14].

### 2.4. Statistical Analysis

The sample size for group comparison was based on available estimations of overweight/obesity in Tuscany and Calabria [4]. Considering a 5% confidence level (alpha error) and 80% statistical power (beta error), the estimated total sample size was at least 281 individuals for both parents and students. Values were expressed as number and percentage for categorical variables or mean ± standard deviation (SD) for continuous variables. Group differences were estimated using χ^2^ tests for categorical variables, or *t*-tests for continuous variables. Multivariable regression analyses were then conducted to investigate the association between dependent and independent variables (exposures). Variables included age, gender, education level, BMI categories, region of residence, compliance with WHO PA recommendations (Yes or No), METs per week, MD score and nutritional knowledge.

IBM SPSS software (version 28.0; SPSS Inc., Chicago, IL, USA) was used for analysis.

## 3. Results

### 3.1. Characteristics of Study Population

Overall, 426 students (233 girls, 54.7%) with an average age of 11.6 ± 1.5 years and 298 parents (222 women, 74.5%) aged 44.5 ± 5.8 years, participated in the study. Overweight/obese participants were 139 (32.6%) among students and 118 (39.6%) among parents, with an adequate average MD score in both groups (30.7 ± 4.2 and 31.1 ± 4.3, respectively). Age-specific WHO suggestions for physical activity were respected by only 164 out of 426 students (38.5%) whereas 194 out of 298 parents (65.1%) followed WHO suggestions.

Descriptive characteristics of the students and parents in the two areas of Italy are shown in Table 1. The BMI of the participants was higher in Southern than in Central Italy for both students and parents (*p* < 0.001 for both). In the Tuscan population, BMI categories showed no gender differences among students, whereas obese parents were more frequently men than women (*p* < 0.05, data not shown). In Southern Italy, both obese students and obese parents were more frequently men than women (*p* < 0.05 for both, data not shown). No geographical differences were observed in the adherence to the MD, although the parents’ score was slightly lower in Calabria than in Tuscany. Students moved less in Calabria than in Tuscany, while there were no regional differences as regards the parents. More precisely, compliance with the WHO recommendations among children was 34% in Calabria against 45% in Tuscany (*p* < 0.025), while compliant parents were 63% and 68%, respectively (ns), with no gender differences. Students showed a lower level of nutritional knowledge at the GeSNK test in Calabria than in Tuscany (Table 1). Conversely, no regional differences were observed among parents (Table 1).

### 3.2. The Influence of Parents

Overall, in a multivariable linear regression analysis the MD score of students was positively influenced by the dietary habits of their parents, although the influence reduced with students’ age (Table 2). More precisely, students’ consumption frequency of fruit and legumes was positively associated with their parents’ consumption (Table 3).

The consumption of potatoes and legumes by students is also positively influenced by living in Calabria, while the consumption of fruit and fish reduces with age (data not shown).

Likewise, students’ physical activity was positively associated with the physical activity of their parents, independently of the geographical area (Table 4).

The source of the students’ nutritional knowledge was no different between the two regional areas. In particular, both in Tuscany and Calabria parents were reported to be the main source of nutritional knowledge (57.5% and 60.7%, respectively), followed by school teachers (26.8% and 26.7%), the web or the media (21.2% and 23.5%), sports coaches (11.7 and 10.9) and friends (7.3% and 3.6%). The students’ nutritional knowledge was positively influenced by age and regional area (Tuscany) (Table 5).

### 3.3. Interaction between Geographical Area and Family Environment on BMI of Adolescents

The interaction between geographical area, parental characteristics and students’ BMI was then investigated.

The only parental characteristic identified as an independent determinant of the students’ BMI at multivariable analysis (also adjusted for age, gender, PA category, MD category and GeSNK category of students) was parental BMI category (Table 6).

## 4. Discussion

These data shed new light on the regional differences of BMI in Italy, showing that (1) there is a direct relationship between the BMI of children and parents that is independent of a geographical area; (2) the eating or physical activity habits of parents are transmitted to their children even if they do not directly affect the BMI of the children; (3) geographical area is not in itself a determinant of the BMI of students.

Diet and physical activity are among the best-known and studied factors that influence a person’s weight. Adherence to the Mediterranean Diet (MD) and physical activity were reported to reduce the risk of developing cardiovascular risk factors (hypertension, diabetes, dyslipidemia) and chronic non-communicable diseases from childhood onward [23,24,25]. Family environment is therefore now considered an important target for prevention of childhood obesity, which often tracks into adulthood [26]. Excess body fat has been attributed to both high intake of energy-dense, nutrient-poor foods and beverages, such as chips, salted snacks, sweets and soft drinks, and a sedentary lifestyle [27]. The World Health Organization (WHO) indeed recommend associating adherence to the MD with at least 60 min of daily moderate-to-vigorous physical activity for children and adolescents in order to reduce the risks of obesity [28] and to achieve proper psychomotor development [29]. Unfortunately, the MD appears to be followed less and less by younger generations in most Mediterranean countries [30,31], sedentarism is spreading [32,33] and childhood obesity is on the rise. In Europe, childhood obesity has a North to South gradient [4,5], also observed at a national level in Italy [4]. The search for the determinants of these differences is of great interest for prevention strategies.

Parents’ habits are among the main factors that shape the lifestyle habits of their children, influencing their respect for healthy patterns especially at a developmental age [33]. It has, in fact, been reported that the adherence of parents to the MD favorably conditions the eating behavior and food choices of their children [34,35,36,37,38]. It must be said that other studies showed a moderate or weak association in dietary habits, with remarkably varied results [39]. In the present study, the adherence of parents to the MD favorably affected the students’ MD, although an inverse association was observed with age. This pattern may suggest a deviation from family habits during the transition from childhood to adolescence, a stage characterized by marked shifts in cognitive development and an increased sense of autonomy in decision-making about own eating patterns [37]. Thus, combined with evidence that eating behavior is established early in life, these findings suggest that childhood and adolescence are better stages for establishing healthy dietary habits to prevent obesity [40]. In the present study, the amount of physical activity practiced by children was also affected by parents’ habits, as described in previous studies [33]. Parents can influence children’s physical activity through modelling, providing support for physical activity in the home environment and establishing rules or expectations for physical activity [41].

When considering geographical differences, the prevalence of obesity was found to be higher in Calabria, a Southern Italian region, than in Tuscany, in line with recent data [4]. However, in the multivariate regression analysis, the geographical region was not selected as an independent determinant of student obesity, even though Tuscan children moved more than Calabrian children. In contrast to a previous study in Italy [42], no regional differences in MD habits were found.

Importantly, in the present study adherence to the MD and physical activity do not emerge as determinants of childhood obesity. This observation agrees with a recent systematic review where an inverse association of MD adherence with BMI values or the prevalence of overweight and obesity was observed in only 10/26 papers investigating children and/or adolescents [43]. Exploring the relationship between the MD and overweight/obesity is indeed complex. Cross-sectional design studies do not permit the inference of causal relationships between MD adherence and anthropometric variables. Secondly, BMI is not a clear indicator of abdominal adiposity or fat mass. Finally, self-reported measurement studies could have been influenced by the fact that obese individuals are more likely to under-report their body weight.

To the best of our knowledge, this is the first study to explore the relationship between nutritional knowledge and obesity in adolescents and their parents. Interestingly, there appears to be a discrepancy between the level of nutritional knowledge and obesity in adults and students. More precisely, the lower nutritional knowledge of students in Calabria than in Tuscany was closely associated with a higher degree of obesity. Conversely, regional differences in obesity were independent from nutritional knowledge among adults. Although proper knowledge of nutrition does not necessarily translate into a healthier BMI [44], other cultural descriptors, such as the level of education of parents, could help to better explain these findings. The influence of regional differences on students’ BMI indeed disappears when the education level of parents, the only socioeconomic indicator available in this study, is introduced into the model. At this point the only variable that continues to influence students’ obesity is the phenotype of the parents. If parents are obese, children are more likely to also be obese. In the context of the family environment, socio-cultural aspects were found to play a key role; children from low socioeconomic status were reported to be at the highest risk of obesity [45]. According to Nau et al. [46], social factors mainly operate through physical activity and dietary factors. In Italy, regional differences exist in the availability of sports facilities and of trained personnel for various sports; in the amount of caloric intake; in the level of education of the parents, which was observed to be different in the two populations [47]. The combination of these factors affects the BMI of both children and parents. In conclusion, even though the transmission of virtuous habits appeared to occur from parents to children, parent–child BMI remained the only strong association found. Parental obesity can therefore be the point of convergence of the complex interactions between parents’ and children’s habits and should be one of the most important factors to look for.

This study has several potential limitations. First, this study does not allow for a clear analysis of the role of socio-economic factors. In fact, it was not possible to use the income parameter in a study with these characteristics. The level of education is not a reliable proxy in countries such as Italy where the degree of education is not necessarily associated with income. Second, the participation rate was low although in line with current reports. Third, biases inherent in the use of referred data, such as anthropometry, eating habits and level of physical activity, are possible. Fourth, the use of a convenience sample and the small number of schools examined do not allow us to generalize our results. Studies based on large random samples that are representative of the Italian adolescent population are obviously warranted. Finally, the Mediterranean Diet score investigates the frequency of food consumption by disregarding the total amount of food consumed and excluding other factors that may affect childhood obesity such as snack food and sweetened beverages.

## 5. Conclusions

Childhood obesity is a crucial issue in modern times and prevention strategies need to be improved. Eating habits and physical activity are among the major determinants of BMI, but the way they act in influencing weight status underlies complex interactions with the family environment and the parents’ cultural pattern.

## Figures and Tables

**Table 1:** Characteristics of students and parents according to geographical area of residence.

	Students			Parents		
	Tuscany	Calabria	*p* < *	Tuscany	Calabria	*p* < *
	(n = 179)	(n = 247)		(n = 113)	(n = 185)	
Age (years)	10.9 ± 1.9	12.0 ± 1.1	0.05	45.2 ± 6.3	44.1 ± 5.4	ns
Female gender, n (%)	101 (56%)	132 (53%)	ns	78 (69%)	144 (78%)	ns
BMI (kg/m^2^)	18.3 ± 3.3	20.3 ± 3.8	0.05	23.4 ± 3.1	25.4 ± 4.1	0.05
BMI categories, n (%)						
Underweight	19 (10.6%)	4 (1.6%)		10 (8.8%)	0 (0.0%)	
Normal weight	119 (66.5%)	145 (58.7%)		74 (65.5%)	96 (51.9%)	
Overweight	22 (12.3%)	56 (22.7%)		26 (23.0%)	69 (37.3%)	
Obese	19 (10.6%)	42 (17.0%)	0.001	3 (2.7%)	20 (10.8%)	0.001
MD score (units)	30.8 ± 3.8	30.7 ± 4.5	ns	32.5 ± 3.7	30.3 ± 4.4	0.05
METs total (METs)	3530 ± 3135	2891 ± 3762	ns	2258 ± 1849	2762 ± 2594	ns
Physical activity categories, n (%)						
Inactive	18 (10%)	47 (19%)		21 (19%)	43 (23%)	
Moderate	68 (38%)	101 (41%)		56 (50%)	67 (36%)	
Active	93 (52%)	99 (40%)	0.011	36 (32%)	75 (41%)	ns
WHO compliant, n (%)	80 (45%)	84 (34%)	0.025	77 (68%)	117 (63%)	ns
GeSNK score (units)	55 ± 14	43 ± 10	0.05	66 ± 10	65 ± 6	ns
GeSNK categories, n (%)						
Low knowledge	20 (20%)	122 (49%)		0 (0%)	0 (0%)	
Medium knowledge	41 (41%)	122 (49%)		11 (28%)	27 (15%)	
High knowledge	38 (38%)	3 (1%)	0.001	29 (73%)	154 (85%)	ns

* = at Student’s *t*-test or Chi square test as appropriate. BMI = body mass index; MD = Mediterranean Diet; METs = metabolic equivalents; WHO = World Health Organization; GeSNK = the General and Sport Nutrition Knowledge questionnaire.

**Table 2:** Determinants of students’ Mediterranean Diet (MD) score at multivariate linear regression *.

Exposures	B	(95% CI for B)	*p*
Age	−0.37	(−0.71 to −0.02)	0.036
Sex (women)	−0.07	(−1.06 to 0.92)	0.887
Geographic region (south)	0.91	(−0.31 to 2.12)	0.142
Parent MD Score	0.19	(0.07 to 0.31)	0.002

* dependent variable: students’ MD score, adjusted for variables reported in the Table.

**Table 3:** Association between the consumption frequency of a specific food by the students and the consumption frequency of the same food by the parents at multivariate linear regressions *.

Exposures	B	(95% CI for B)	*p*
Unrefined cereals	0.125	(−0.019 to 0.270)	0.088
Potatoes	0.025	(−0.089 to 0.139)	0.668
Fruit	0.124	(0.001 to 0.249)	0.050
Vegetables	0.111	(1.596 to 0.112)	0.104
Legumes	0.136	(0.005 to 0.267)	0.041
Fish	0.104	(−0.020 to 0.228)	0.100
Red meat	0.026	(−0.073 to 0.125)	0.610
White meat	0.031	(−0.074 to 0.136)	0.563
Dairy products	0.096	(−0.007 to 0.198)	0.067

* adjusted for age, gender, BMI category and geographic region of students.

**Table 4:** Determinants of students’ physical activity at multivariate linear regression *.

Exposures	B	(95% CI for B)	*p*
Age	231.23	(−30.14 to 492.93)	0.082
Sex (women)	415.30	(−334.75 to 1165.35)	0.277
Geographic region (South)	−483.05	(−1389.87 to 422.45)	0.294
Parents’ METs per week	0.24	(0.07 to 0.41)	0.006

* dependent variable: students’ METs per week, adjusted for variables reported in the Table; METs = Metabolic equivalents.

**Table 5:** Determinants of nutritional knowledge of students at multivariate linear regression *.

Exposures	B	(95% CI for B)	*p*
Age	0.34	(0.25 to 0.43)	<0.001
Sex (women)	0.03	(−0.14 to 0.21)	0.710
Geographic region (Calabria)	−0.59	(−0.87 to −0.31)	<0.001
Parents’ GeSNK score percentiles	0.08	(0.05 to 0.37)	0.440

* dependent variable: student GeSNK score percentiles, adjusted for variables reported in the Table; GeSNK = the General and Sport Nutrition Knowledge questionnaire.

**Table 6:** Determinants of students’ body mass index category at univariate and multivariate linear regression analysis *.

	Univariate Analysis			Multivariate Analysis *		
Exposures	B	(95% CI for B)	*p*	B	(95% CI for B)	*p*
Geographic region (south)	0.32	(0.17 to 0.47)	<0.001	0.32	(−0.03 to 0.67)	0.075
Father’s education level	−0.02	(−0.13 to 0.10)	0.780	−0.05	(−0.23 to 0.14)	0.608
Mother’s education level	−0.02	(−0.13 to 0.10)	0.783	0.11	(−0.07 to 0.29)	0.215
Physical activity category	0.01	(−0.12 to 0.14)	0.910	−0.06	(−0.20 to 0.08)	0.370
Parent MD score category	0.05	(−0.15 to 0.25)	0.612	0.04	(−0.18 to 0.25)	0.740
Parent GeSNK category	−0.10	(−0.40 to 0.21)	0.523	−0.18	(−0.47 to 0.12)	0.235
Parent BMI category	0.30	(0.16 to 0.44)	<0.001	0.29	(0.13 to 0.45)	<0.001

* adjusted also for age, gender, physical activity category, MD category and GeSNK category of students. MD = Mediterranean Diet; GeSNK = the General and Sport Nutrition Knowledge questionnaire; BMI = Body Mass Index.

## Data Availability

The data presented in this study are available on request from the corresponding author. The data are not publicly available due to privacy restrictions.

## Supplementary Materials

The following supporting information can be downloaded at: https://www.mdpi.com/article/10.3390/ijerph20021157/s1.
