# Supplementary material for: Interaction between Geographical Areas and Family Environment of Dietary Habits, Physical Activity, Nutritional Knowledge and Obesity of Adolescents
Source: Int J Environ Res Public Health. 2023 Jan 9;20(2):1157. doi: 10.3390/ijerph20021157 (PMC9859590; doi:10.3390/ijerph20021157)
Supplement: Supplementary file 1 [file ijerph-20-01157-s001.zip › ijerph-2059560-supplementary.pdf]

GeNSK- General and Sport Nutrition Knowledge Questionnaire

The purpose of this questionnaire is to identify myths and false nutritional beliefs in order to improve nutritional education programs

Name/Surname:
Gender: Male ☐ Female ☐
Date of Birth:
Weight: Height:
Employment father/mother:

Level of education father/mother:
Do you practice any Sport? YES☐ NO☐
Sport:
How many times a week:
Average length: *(minutes per day)*:

My knowledge about healthy eating are driven by: (you can choose more than one answer)

Nutritional education programs at school	☐	*From what I watch on the TV*	☐
Nutritional education programs in other places	☐	*From the web*	☐
From my teachers	☐	*From my friends*	☐
From my parents	☐	*I have no knowledge about to healthy eating*	☐
From my coaches	☐	*Anything else……………………………………………………*	

SECTION 1: GENERAL NUTRITION

Following are the questions refer to the nutritional composition of some foods. Choose the answer with an X.

1. **The Carbohydrate content of such foods is:**

	High	Low or absent	I do not know
Boiled ham	☐	☐	☐
White bread	☐	☐	☐
Tomato	☐	☐	☐
Apple	☐	☐	☐
Ricotta	☐	☐	☐
Breakfast cereals	☐	☐	☐

2. **The Protein content of such foods is:**

	High	Low or absent	I do not know
Chicken meat	☐	☐	☐
Dried beans	☐	☐	☐
Pear	☐	☐	☐
Rice	☐	☐	☐
Codfish	☐	☐	☐
Parmesan	☐	☐	☐
Chocolate	☐	☐	☐

3. **The Fat content of such foods is:**

	High	Low or absent	I do not know
Salami	☐	☐	☐
Mayonnaise	☐	☐	☐
Dried chickpeas	☐	☐	☐
Pasta	☐	☐	☐
Butter	☐	☐	☐
Jam	☐	☐	☐

4. **The Fiber content of such foods is:**

	High	Low or absent	I do not know
Honey	☐	☐	☐
Brown bread	☐	☐	☐
Chicken broth	☐	☐	☐
Potatoes	☐	☐	☐
Pear	☐	☐	☐
White bread	☐	☐	☐

5. **The Salt content of such foods is:**

	High	Low or absent	I do not know
White bread	☐	☐	☐
Courgettes	☐	☐	☐
Canned peas	☐	☐	☐
Canned tuna	☐	☐	☐
Frozen peas	☐	☐	☐

6. **The Calcium content of such food is:**

	High	Low or absent	I do not know
Turkey breast	☐	☐	☐
Peas	☐	☐	☐
Walnut	☐	☐	☐
Olive oil	☐	☐	☐
Brown bread	☐	☐	☐

7. **The Iron content of such foods is:**

	High	Low or absent	I do not know
Calf meat	☐	☐	☐
Apple	☐	☐	☐
Honey	☐	☐	☐
Sea bass	☐	☐	☐

8. **The Potassium content of such foods is:**

	High	Low or absent	I do not know
Pasta	☐	☐	☐
Dried lentils	☐	☐	☐
Olive oil	☐	☐	☐
Honey	☐	☐	☐

Are these claims true or false? (only one answer is possible)

**9**	*The egg white is high in cholesterol*		
	**TRUE** ☐	**FALSE** ☐	**I DO NOT KNOW** ☐
**10**	*The high-fat meals are ever high in cholesterol*		
	**TRUE** ☐	**FALSE** ☐	**I DO NOT KNOW** ☐
**11**	*The olive oil is high in monounsaturated fat*		
	**TRUE** ☐	**FALSE** ☐	**I DO NOT KNOW** ☐
**12**	*The dried fruit is a good source of essential fatty acids*		
	**TRUE** ☐	**FALSE** ☐	**I DO NOT KNOW** ☐
**13**	*The ripened cheese are saltier than the fresh one*		
	**TRUE** ☐	**FALSE** ☐	**I DO NOT KNOW** ☐
**14**	*An high-energy food is exclusively a fat food*		
	**TRUE** ☐	**FALSE** ☐	**I DO NOT KNOW** ☐
**15**	*The brown bread is richer in fiber than the white one*		
	**TRUE** ☐	**FALSE** ☐	**I DO NOT KNOW** ☐
**16**	*Bran is the outer part of the grain kernel that is very high in fiber*		
	**TRUE** ☐	**FALSE** ☐	**I DO NOT KNOW** ☐
**17**	*Tinned pulses are saltier than the dry one*		
	**TRUE** ☐	**FALSE** ☐	**I DO NOT KNOW** ☐
**18**	*Omega-3 and omega-6 are particular fatty acids*		
	**TRUE** ☐	**FALSE** ☐	**I DO NOT KNOW** ☐
**19**	*Our body creates vitamin D from direct sunlight on our skin when we are outdoors*		
	**TRUE** ☐	**FALSE** ☐	**I DO NOT KNOW** ☐
**20**	*The iron in meat are more easily absorbed than the same mineral found in vegetables*		
	**TRUE** ☐	**FALSE** ☐	**I DO NOT KNOW** ☐
**21**	*A variety of foods contain a natural amount of sodium*		
	**TRUE** ☐	**FALSE** ☐	**I DO NOT KNOW** ☐
**22**	*Dairy are a good iron source*		
	**TRUE** ☐	**FALSE** ☐	**I DO NOT KNOW** ☐
**23**	*Carrots are a good source of vitamin A*		
	**TRUE** ☐	**FALSE** ☐	**I DO NOT KNOW** ☐

24. The glycemic index food: (only one answer is possible)

indicates the foods carbohydrates content	☐
indicates the food's effect on a person's blood glucose	☐
indicates the food's effect on a person's blood glucose after the ingestion of protein	☐
indicates the foods energy density	☐

Are these claims true or false? (only one answer is possible)

**25**	*An unbalanced diet is the only risk factor for the development of cardiovascular disease*		
	**TRUE** ☐	**FALSE** ☐	**I DO NOT KNOW** ☐
**26**	*In the obesity the diet play an important role, physical activity no*		
	**TRUE** ☐	**FALSE** ☐	**I DO NOT KNOW** ☐
**27**	*A low calcium and vitamin D intake during life, associated with a lack of physical activity may increase fracture risk*		
	**TRUE** ☐	**FALSE** ☐	**I DO NOT KNOW** ☐
**28**	*The fiber helps to ease constipation*		
	**TRUE** ☐	**FALSE** ☐	**I DO NOT KNOW** ☐
**29**	*To obtain a healthy weight loss the carbohydrates must not be removed from the diet*		
	**TRUE** ☐	**FALSE** ☐	**I DO NOT KNOW** ☐

SECTION 2: SPORT NUTRITION

Are these claims true or false? (only one answer is possible)

**30**	*To eat carbohydrate is no good for an athlete*		
	**TRUE** ☐	**FALSE** ☐	**I DO NOT KNOW** ☐
**31**	*B-group vitamins play an important role in muscle metabolism*		
	**TRUE** ☐	**FALSE** ☐	**I DO NOT KNOW** ☐
**32**	*Athletes must reduce the fat intake to a minimum*		
	**TRUE** ☐	**FALSE** ☐	**I DO NOT KNOW** ☐
**33**	*Consuming carbohydrates after 5 pm can enhance performance*		
	**TRUE** ☐	**FALSE** ☐	**I DO NOT KNOW** ☐
**34**	*Eating more protein will make muscles bigger*		
	**TRUE** ☐	**FALSE** ☐	**I DO NOT KNOW** ☐
**35**	*For a sporty person, the daily intake should not include more than 15% of fats*		
	**TRUE** ☐	**FALSE** ☐	**I DO NOT KNOW** ☐
**36**	*Athletes can eat whatever they want because they have a fast metabolism*		
	**TRUE** ☐	**FALSE** ☐	**I DO NOT KNOW** ☐
**37**	*Physical exercise is the main factor improving muscular strength*		
	**TRUE** ☐	**FALSE** ☐	**I DO NOT KNOW** ☐
**38**	*An excessive dietary protein intake can lead to liver and kidney damage*		
	**TRUE** ☐	**FALSE** ☐	**I DO NOT KNOW** ☐
**39**	*An athlete must have a meal at the end of the training session*		
	**TRUE** ☐	**FALSE** ☐	**I DO NOT KNOW** ☐
**40**	*To reduce pasta, potato and bread intake during the training period is needed*		
	**TRUE** ☐	**FALSE** ☐	**I DO NOT KNOW** ☐
**41**	*A man and woman of the same age, practicing the same sport have the same energy requirements*		
	**TRUE** ☐	**FALSE** ☐	**I DO NOT KNOW** ☐
**42**	*It is advisable for an athlete eating a low glycemic index meal but rich in carbohydrates, 1-2 hours after training*		
	**TRUE** ☐	**FALSE** ☐	**I DO NOT KNOW** ☐
**43**	*Athletes practicing extensive training have double protein requirement than the general population*		
	**TRUE** ☐	**FALSE** ☐	**I DO NOT KNOW** ☐
**44**	*Drinking fluids before, during and after a competition is needed*		
	**TRUE** ☐	**FALSE** ☐	**I DO NOT KNOW** ☐
**45**	*Coaches must not allow drinking fluid during a training*		
	**TRUE** ☐	**FALSE** ☐	**I DO NOT KNOW** ☐
**46**	*The best advice for athletes is to drink when they are thirsty*		
	**TRUE** ☐	**FALSE** ☐	**I DO NOT KNOW** ☐
**47**	*For an athlete, cold water quenches thirst better*		
	**TRUE** ☐	**FALSE** ☐	**I DO NOT KNOW** ☐
**48**	*Athletes can use ice cube to quench their thirst during training*		
	**TRUE** ☐	**FALSE** ☐	**I DO NOT KNOW** ☐
**49**	*A great deal to support performance in athletes is water*		
	**TRUE** ☐	**FALSE** ☐	**I DO NOT KNOW** ☐

**50**	*Sports drinks and energy drinks are the same things*		
	**TRUE** ☐	**FALSE** ☐	**I DO NOT KNOW** ☐
**51**	*Sports drinks contain minerals*		
	**TRUE** ☐	**FALSE** ☐	**I DO NOT KNOW** ☐

52.What is the most appropriate beverage after two hours of training?

Energy drink	☐
Sports drink	☐
Fruit juice	☐
Cola	☐
I do not know	☐

Are these claims true or false? (only one answer is possible)

**53**	*For athletes, supplements can be used as valid meals replacement*		
	**TRUE** ☐	**FALSE** ☐	**I DO NOT KNOW** ☐
**54**	*For athletes, it is impossible to reach the iron requirement they need supplements*		
	**TRUE** ☐	**FALSE** ☐	**I DO NOT KNOW** ☐
**55**	*Sports drinks contain caffeine*		
	**TRUE** ☐	**FALSE** ☐	**I DO NOT KNOW** ☐
**56**	*If athletes are not able to fulfill their proteins requirement, they need to take amino acids supplements*		
	**TRUE** ☐	**FALSE** ☐	**I DO NOT KNOW** ☐
**57**	*Supplements are necessary both in professional and recreational sports*		
	**TRUE** ☐	**FALSE** ☐	**I DO NOT KNOW** ☐
**58**	*Food supplements are safe so it is right to use them without any expert advice*		
	**TRUE** ☐	**FALSE** ☐	**I DO NOT KNOW** ☐
**59**	*In strength sports, vitamin c supplements are ever required*		
	**TRUE** ☐	**FALSE** ☐	**I DO NOT KNOW** ☐
**60**	*Unseasoned meals are the best choice for muscle building and toning*		
	**TRUE** ☐	**FALSE** ☐	**I DO NOT KNOW** ☐
**61**	*Meat and egg white contain protein, other foods not. This is the foundation of the athlete's nutrition*		
	**TRUE** ☐	**FALSE** ☐	**I DO NOT KNOW** ☐
**62**	*Athletes can eat "light" products at will*		
	**TRUE** ☐	**FALSE** ☐	**I DO NOT KNOW** ☐
